# The Effect of the Sodium to Potassium Ratio on Hypertension Prevalence: A Propensity Score Matching Approach

**DOI:** 10.3390/nu8080482

**Published:** 2016-08-06

**Authors:** Junhyung Park, Chang Keun Kwock, Yoon Jung Yang

**Affiliations:** 1 Nutrition and Diet Research Group, Korea Food Research Institute, Seongnam-Si, Kyunggi-Do 13539, Korea; urittang@naver.com; 2 Department of Foods and Nutrition, College of Natural Sciences, Dongduk Women’s University, Seoul 02748, Korea; yjyang@dongduk.ac.kr

**Keywords:** sodium to potassium ratio, hypertension prevalence, blood pressure, selection bias, propensity score matching, logistic regression, average treatment effect on the treated subjects

## Abstract

This study investigated the effect of the sodium to potassium ratio on hypertension prevalence and blood pressure. The study population was constructed by pooling the Korean National Health and Nutrition Examination Surveys between 2010 and 2014. The study population was divided into quartiles based on the sodium to potassium ratio, and the effect was inferred by the difference in hypertension prevalence across quartiles by six pairwise comparisons using a propensity score matching technique. The quartiles with the higher sodium to potassium ratio had higher hypertension prevalence rates based on the following pairwise comparisons: the first vs. third quartile, the first vs. fourth quartile, the second vs. third quartile, and the second vs. fourth quartile. The prevalence differences were 2.74% point (*p* < 0.05), 3.44% point (*p* < 0.01), 2.47% point (*p* < 0.05), and 2.95% point (*p* < 0.01), respectively. In addition, statistically significant higher systolic (*p* < 0.05) and diastolic blood pressure (*p* < 0.01) was observed in the second quartiles compared to the first quartiles. Because a strong association was also detected between the sodium to potassium ratio and blood pressure even at a low level of sodium to potassium ratio, a lower sodium to potassium ratio diet than a usual diet is recommended to control high blood pressure in Korea.

## 1. Introduction

The hypertension prevalence rates of Korean male and female adults aged 30 years or above are 32.4% and 22.2% in Korea [1], respectively. Hypertension alone claims two trillion Korean won, which amounts to approximately two billion U.S. dollars, in medical expenses annually [2]. Therefore, hypertension causes some of the heaviest social burdens through medical insurance and personal suffering. Hypertension attracts attention as a strong risk factor for cardiovascular diseases and stroke, which are responsible for the highest mortality rates in Korea [1]. Sodium intake is a significant risk factor of hypertension and has been the target of health policies to reduce hypertension prevalence. Many countries have performed public health measures to reduce sodium intake, and these measures are based upon the association between sodium intake and hypertension.

The 2013 Institute of Medicine (IOM) report acknowledged the overall association between sodium intake and blood pressure based on previous studies. However, individual studies do not show a consistent relationship between sodium intake and blood pressure [3]. Therefore, they do not support the existing upper limit of sodium intake (2300 mg per day) and declared not to set the limit because there is no sufficient population based health outcome evidence [3]; though, they acknowledge the overall association between sodium intake and blood pressure based on previous studies. Some studies extended the conclusion of the IOM report and suggested that both lower and higher sodium intake were related with an increased risk of cardiovascular disease compared to the usual sodium intake of 3000–5000 mg per day [4,5]. In response, the U.S. Centers for Disease Control and Prevention (US CDC) and the American Heart Association (AHA) dissociated themselves from the 2013 report [6]. AHA and the US CDC criticized that this line of study reporting a J- or U-shaped relationship was due to the use of an incorrect dietary measure of the sodium intake variable and the improper treatment of statistical problems, such as reverse causality, and confounding in association and in residuals [7]. This conflict between institutions ignited a heated debate over the proper daily sodium intake limits and increased confusion among the public over sodium intake.

He, Li, and MacGregor [8] concluded that a reduction in sodium intake reduced blood pressure among hypertensive and normotensive groups. Their conclusion was drawn from the review of 37 random clinical trials that observed sodium intake and blood pressure for at least four weeks. Based on the authors’ reanalysis of 37 cases, only 56.8% (21 of the cases) reported a statistically significant correlation between sodium intake and blood pressure.

Observational studies carried out in various countries also show very inconsistent results. For instance, Du et al. [9] found statistically significant correlations between sodium intake and hypertension incidence and between the sodium to potassium (Na:K) ratio and hypertension incidence based on a Chinese cohort study. Mente et al. [10] reported a statistically significant positive correlation between urinary sodium excretion and blood pressure from an 18 country cross-sectional study. Zhang et al. [11] also reported a positive correlation between sodium intake and blood pressure from a cross-sectional study consisting of American adults older than 20 years of age who were currently not taking hypertension medications. Huggins et al. [12] found a statistically significant difference in hypertension prevalence only between the lowest and highest quintiles of the Na:K ratio from a cross-sectional study consisting of 50–75 years old Australians. Park et al. [13] failed to find a correlation between sodium intake and blood pressure from a cross-sectional study consisting of young and middle-aged adult Koreans. Ducher et al. [14] did not find a correlation between sodium intake and blood pressure from a small scale cohort study consisting of healthy French subjects. Finally, Kim et al. [15] found that urinary sodium and the Na:K ratio had a significant relationship with blood pressure only among males based on a cross-sectional study consisting of healthy adult Koreans.

The inconsistencies in the results of observational studies reflect the necessity for further evidence regarding the association between Na:K ratio and blood pressure, because widely varying results of observational studies regarding the association between sodium intake and blood pressure or hypertension status could be due to the incorrect measurement of sodium intake and the improper statistical analyses [7]. Additionally, tests are required to determine whether the association between sodium intake and blood pressure is strong enough to affect hypertension prevalence or incidence. Very limited studies have estimated the quantitative hypertension risk of sodium intake. According to the authors’ re-analyses of 23 studies reviewed by Perez and Chang [16], there were only three studies which provided the quantitative risk of the Na:K ratio.

The objective of this study is to examine the effect of the Na:K ratio on hypertension prevalence. The effect of the Na:K ratio was examined because previous studies found that the joint effect of low sodium and high potassium intake on blood pressure may be larger than the effects of either sodium or potassium alone [16,17,18], and some studies also found that the Na:K ratio may affect the pathogenesis of hypertension independent of cardiovascular risk factor [19]. Another reason to look into the Na:K ratio is to avoid the over-adjustment issue caused by the potassium intake variable, which is highly correlated with sodium intake. The hypertension model without the potassium intake variables faces an under-adjustment problem because the potassium intake has an influence on blood pressure through the increase in urinary sodium excretion and the widening of the blood vessels [20,21].

In pursuing the objective, the authors resolve the statistical issues raised by the American Heart Association Science Advisory including reverse causality, residual confounding due to incorrect adjustments, and covariate balances across different Na:K ratio groups [22]. To mitigate the reverse causality issue, subjects with chronic diseases, such as chronic renal failure, diabetes, cardiovascular diseases, stroke, and dyslipidemia were excluded to prevent the influence of chronic diseases on sodium intake. Based on these statistical issues, propensity score matching technique was employed to infer the quantitative effect of the Na:K ratio on hypertension prevalence and blood pressure.

## 2. Data and Methods

### 2.1. Study Population

This study used five years of data from the Korea National Health and Nutrition Examination Survey (KNHANES) collected by the Korea Center for Disease Control and Prevention. The study population consists of 30,206 subjects aged between 20 and 79 years from KNHANES data pooled form 2010 to 2014. Subjects with chronic diseases, such as chronic renal failure, diabetes, cardiovascular diseases, stroke, and dyslipidemia were excluded from the study population to mitigate the reverse causality problem, because strict diets were recommended by physicians to patients with the above chronic diseases. The concern over reverse causality due to hypertension can be eased because the prevalence rates of the lower Na:K ratio groups (Q1 and Q2) were actually lower than the study population average and those of the higher ratio groups, as shown in Table 1 and Table 2. KNHANES collects health-related data through actual measurements or face to face interviews. Weight, height, and blood pressure are measured data, whereas family history of hypertension, drinking, smoking status variables, etc., are collected through face to face interviews by trained interviewers. Details of the KNHANES and its food intake survey were described in the previous studies [23,24].

**Figure 1:**
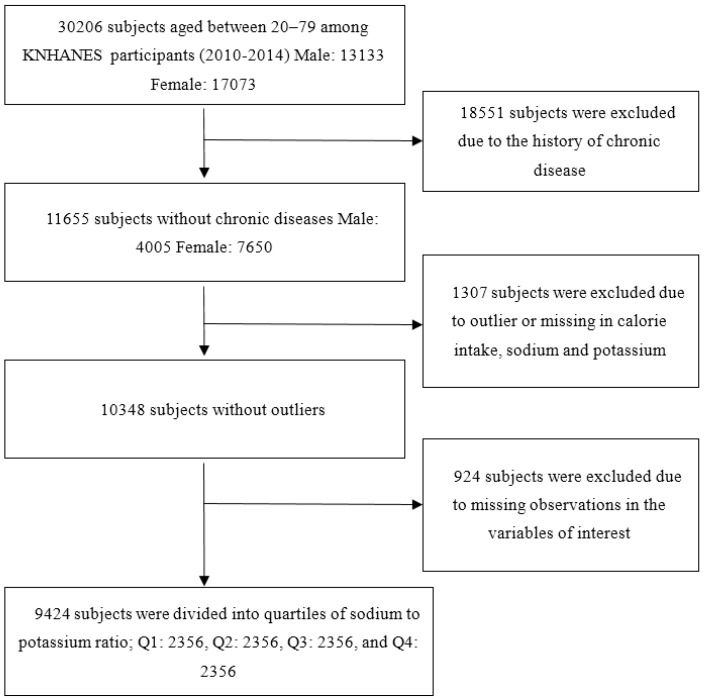
Study population flow.

Since the sodium and potassium intake data are calculated from the 24 h recall food intake survey, too little or too much calorie intake could signal an incorrect food intake survey. Therefore, it is necessary to remove the observations with extreme calorie intake values to control the outliers. Following Kwock and Park [25], intakes below 400 kcal and above 6000 kcal per day were used as the lower limit and upper limit of calorie intake, respectively. The lower limits used in the previous studies ranged from 350 to 800 kcal per day [26,27,28,29], and the upper limits range from 3500 to 8000 kcal per day [26,27,28,29]. Thus the lower and upper limits used in this study are in the range. Due to potential influence from outliers, 112 subjects who reported energy intakes outside of the range were excluded, and 1195 subjects who had missing observations in intake variables were also removed. Additionally, 924 subjects who had missing observations for the variables used in the analysis were removed. Finally, a data set with 9424 subjects was used in this study. The flow of study subjects is shown in the Figure 1. This study was exempt from review by the Institutional Review Board at the Korean National Institute for Bioethics Policy (Project number: P01-201408-RW-03-00).

### 2.2. Study Measures

This study used the propensity score matching technique to estimate the approximate effects of the Na:K ratio on hypertension prevalence. The matching technique was employed because it could resolve the issue of selection bias caused by the determination of membership between the control and treatment groups in observational studies [30]. Selection bias is the systematic difference in the covariate means for the control and treatment groups. Selection bias in the context of this study appears as large mean differences in the mean age, gender ratio, etc. when the subjects are divided into groups based on their Na:K ratios. If the outcomes and hypertension prevalence are compared without fixing the selection bias problem, the estimated outcome difference can be biased in small samples and inconsistent in large samples [31]. Traditional regression approaches, such as logistic regression, are not sufficient to achieve balance between compared groups [30]. Thus, this approach is particularly useful for observational studies in which randomization of the subjects is not feasible [32].

In addition to hypertension risk factors, various socio-demographic variables, such as age, education, income, etc. were included in the hypertension models. Some of these socio-demographic variables affect other covariates as well as the dependent variable. For example, an age variable, which is a significant risk factor of hypertension, is a confounding variable since it can also influence the treatments and the Na:K ratios through the insensitivity of salty taste from aging. Because these confounding variables can influence the direction of the association between the Na:K ratio and hypertension prevalence, they need to be adjusted properly [30]. The propensity score matching technique can fix the confounding problems by achieving very close values of the confounders through matching to the subjects in the treatment group [31].

The basic idea behind propensity score matching is to find a group of untreated subjects similar to the treated subjects in terms of the subject characteristics. However, since finding a subject with similar characteristics increases in difficulty as the number of subject characteristics increases, Rosenbaum and Rubin [33] suggested the use of a propensity score, which is the conditional probability of being a member of the treatment group.

The propensity score is usually estimated with a conventional probit or logistic function. The dependent variable has a value of “1” if a subject belongs to the treatment group; otherwise, the value is “0”. The variables that influence the outcome and treatment variables are used as the covariates. Once the propensity score is estimated, the subject who has the closest propensity score in the control group is matched with a subject in the treatment group using matching algorithms. Balance is achieved between the control and treatment groups during this matching process. Because the distributions of covariates that characterize the subjects in the control and treatment groups are more similar after matching than the random sample, a correct treatment effect can be obtained [32]. The most popular matching algorithms are nearest-neighborhood matching, radius matching, and kernel matching [34].

The matching quality is checked after matching by testing the balances of the covariates in the control and treatment groups. Then, a t-test is used to test the equality of the covariate means across the control and treatment groups. If the matching was executed properly, the null hypothesis of equal means should not be rejected. The percent standardized bias should also be checked for each covariate, and 5% should be sufficient [35,36]. Sianesi [37] suggests that the propensity score should be re-estimated with matched subjects only, and the pseudo-R^2^ values should be compared to assess the overall balance of the covariates. Then, the pseudo-R^2^ should be very low since the covariates are similar in both control and treatment groups.

In random clinical trials, the outcome difference between the control and treatment groups is the treatment effect because the subjects are randomized. Although cross-sectional studies cannot determine rigorous causal effect, the propensity score matching technique can provide an approximation of the treatment effect [32]. Because the randomization of subjects is almost achieved between the control and treatment groups with matching, the estimates of average treatment effect on the treated subjects (ATT) are obtained by comparing the mean outcomes between the control and treatment groups [34]. Again, the significance of the ATT is tested using a t-test since this test is about the equality of the two-sample means.

### 2.3. Statistical Analyses

The specific objective of this study was to test the significance of differences in hypertension prevalence across different Na:K ratio levels. For this objective, the subjects were divided into quartiles based on their Na:K ratio levels, and then the means of the hypertension prevalence of the quartiles were compared. Instead of comparing four (N) treatments with different Na:K ratios at the same time, six (N × (N − 1)/2) different pairwise comparisons were performed because they were practical alternatives [38]. For the pairwise comparisons, the groups with the higher Na:K ratios were assigned as the treatment groups, and the conditional probability of being a member of a treatment group was estimated. For example, the conditional probability of being a member of Q2 had to be estimated first for the pairwise comparison between Q1 with a Na:K ratio of 0.74 and Q2 with a Na:K ratio of 1.21. Then, the difference in the hypertension prevalence between the groups could be calculated after matching.

The probability function used to estimate the conditional probability of being a member of a treatment group is a logistic function: its dependent variable has a value of “1” if the subject is the member of treatment group and “0” otherwise. Covariates used in the logistic function were age, sex, body mass index (BMI: kg/m^2^), marital status, monthly household income, education in years, walking hours per day, smoking status, drinking status, stress level, use of nutritional labels, family history of hypertension, etc. The definitions of the variables used to estimate the propensity score are shown in Table 1. The variables selected in the model are generally related to the Na:K ratio or the hypertension outcome since only variables affecting the treatment or outcome should be included [39]. However, there are no general rules about what to include. Some of the variables, such as monthly household income and the use of nutritional labels, did not have significantly different means across the quartiles, but they were included because they were important variables theoretically. The propensity scores were estimated using the same procedure with identical covariates for all pairwise comparisons as follows: Q1 vs. Q2, Q1 vs. Q3, Q1 vs. Q4, Q2 vs. Q3, Q2 vs. Q4, and Q3 vs. Q4.

The matching procedures were conducted based on the estimated propensity scores from the logistic regressions using the nearest-neighborhood matching algorithm, radius matching algorithm, and kernel matching algorithm. Because different matching algorithms resulted in almost identical results in the ATT, we reported the results obtained by 1:1 nearest-neighborhood matching with caliper 0.01 without the replacement method using STATA 12.0 (STATACorp, College Station, TX, USA). The common support restriction was used, and the observations in the range between the minimum and maximum propensity scored of treatment group were used. The observations outside of this range were excluded.

After matching, the balance was confirmed by a two-sided t-test to determine the equality of the sample means of the control and treatment groups for each covariate. The percent biases were also evaluated for every covariate to determine whether they were within the 5% limit. Finally, pseudo-R2 values for all pairwise comparisons were assessed to determine whether the overall balance of the covariates was satisfied.

Once balance was achieved between the control and treatment groups, the difference in the hypertension prevalence rates in the two groups was the approximation of the treatment effect, and a *t*-test was used to test the significance of the effect. A one-sided *t*-test was conducted for each pairwise comparison to test the null hypothesis; the groups with the higher Na:K ratios had the higher hypertension prevalence.

All statistical analyses (i.e., data management to construct the study population, estimation of logistic regression for the propensity score, matching, and significance test on ATT) were performed with STATA Release 12 [40].

## 3. Results

### 3.1. General Characteristics of the Study Population

Table 1 provides the general characteristics of the study population and the definitions of the variables used to estimate of the propensity score. The average sodium intake was 4533.17 ± 30.24 mg/day, and potassium intake was 3104.64 ± 15.97 mg/day. The average sodium intake was well above the World Health Organization (WHO) daily recommendation of 2000 mg/day, whereas the average potassium intake was approximately 88% of the WHO daily recommendation [41]. Therefore, the average sodium to potassium ratio was 1.54 ± 0.01.

Overall, the average hypertension prevalence was 19.56%, which was relatively low. The low prevalence was found because subjects with hypertension as a comorbidity of other chronic diseases were excluded. The average BMI was 22.87 ± 0.03; this was also affected by the exclusion of obese subjects with chronic diseases. The average age was approximately 46 years, and the proportion of females was 68.19%. The proportions of smokers and drinkers were lower due to the high female to male ratio.

Table 2 shows the general characteristics of subjects by Na:K ratio quartiles and the potential confounding variables. None of the quartiles met the upper limit of sodium intake (2000 mg/day), however, only Q1 was above the daily recommendation for potassium. Because there were large differences in the average proportions of females and manual workers and smoking and drinking statuses across quartiles, the presence of selection bias was highly suspected and the use of the propensity score matching technique was warranted. Other variables, such as age, marital status, education, etc., also needed to be controlled due to the large differences in sample means across quartiles.

Q1 shows a low intake of calories and fat and a high intake of fiber, potatoes, and fruits which are major the sources of potassium, as shown at the bottom of Table 2. Although vegetable consumption is a major source of potassium, Q1 was low in vegetable intake, because Koreans with a higher sodium intake typically consume a large amount of pickled vegetables, which are very salty. These data do not differentiate between fresh and pickled vegetables.

### 3.2. The Effect of the Sodium to Potassium Ratio on Hypertension Prevalence

Table 3 reports the estimated ATTs for six pairwise comparisons using propensity score matching and includes the hypothesis test results for the significance of the differences in hypertension prevalence. After matching, the hypertension prevalence of Q1 was 17.13% and Q3 was 19.87% based on the pairwise comparison between Q1 and Q3. The difference in the hypertension prevalence (2.74% point) was significant (*p* < 0.05) based on the one-sided t-test. This result favors the hypothesis that the group with the higher Na:K ratio has higher hypertension prevalence. In the pairwise comparison between Q1 and Q4, Q4 had a higher hypertension prevalence by 3.44% point (*p* < 0.01). In the pairwise comparisons between Q2 and Q3 and between Q2 and Q4, the groups with higher Na:K ratios had a higher hypertension prevalence by 2.47% point (*p* < 0.05) and 2.95% point (*p* < 0.01), respectively.

Four pairwise comparisons, Q1 vs. Q3, Q1 vs. Q4, Q2 vs. Q3, and Q2 vs. Q4, showed higher hypertension prevalence in the higher Na:K ratio groups. The pairwise comparisons Q1 vs. Q2 and Q3 vs. Q4 did not show any differences in hypertension prevalence.

Table 3 also shows the sodium and potassium intakes after matching for pairwise comparisons across quartiles of the Na:K ratio. The subjects in Q1 consumed an average of 2588.94 mg/day and 3641.10 mg/day of sodium and potassium, respectively whereas the subjects in Q2 consumed an average of 3692.91 mg/day and 3050.27 mg/day of sodium and potassium, respectively. Based on the rigorous statistical tests, there was no difference in hypertension prevalence between Q1 and Q2. Therefore, the subjects in Q2 may consume up to 4405.73 mg/day of sodium without raising their hypertension prevalence, if they consumed the same amount of potassium as the subjects in Q1 and held their sodium to potassium ratio to 1.21. However, because a strong correlation was detected between the Na:K ratio and blood pressure in the comparison of Q1 and Q2, 4405.73 mg/day of sodium intake could be hazardous to blood pressure.

The first significant difference in hypertension prevalence was from the comparison of Q1 and Q3. The subjects in Q1 had an average intake of 2610.23 mg/day of sodium and 3641.29 mg/day of potassium, whereas the subjects in Q3 had an average intake of 4628.49 mg/day of sodium and 2833.01 mg/day of potassium. The hypertension prevalence rates were 17.13% and 19.87% for Q1 and Q3, respectively. In this pairwise comparison, the effect of one unit change in Na:K ratio on the hypertension ratio is 3.08% point.

Table 4 shows the balance check results of covariates used in the estimation of propensity scores before and after matching. None of the t-tests assessing the equality of covariate means in the control and treatment groups rejected the null hypothesis after matching. All of the percent bias statistics were below 5% except the smoking variable between Q2 and Q4. Even though the smoking variable remained unbalanced at 5% bias criterion after matching, the percent bias approach does not have a clear standard for the success of the matching [36], and the over-all balance results and pseudo-R^2^ show that there were no significant differences in matched samples. The pseudo-R^2^ values obtained from the re-estimation of the propensity score with only matched data were all very low, and some were close to 0.01, as shown at the bottom of Table 4. These results show that over-all and covariate specific balances were achieved by matching and that the quality of the matching was satisfactory.

### 3.3. The Effect of the Sodium to Potassium Ratio on Blood Pressure

To examine the effect of the Na:K ratio on systolic (SBP) and diastolic blood pressure (DBP), further propensity score matching analyses were performed. For this purpose, all subjects who were previously diagnosed and currently taking hypertension medications were excluded from the dataset used in the above pairwise comparisons. This exclusion removed any remaining possibility of reverse causality after the exclusion of chronic patients. The propensity scores were estimated and matched again after the exclusion, and the differences in mean SBP and DBP were estimated for all six pairwise comparisons. Table 5 reports the average treated effects of Na:K ratio on blood pressure and the significance of ATTs from the six pairwise comparisons after excluding hypertension patients.

Three pairwise comparisons, Q1 vs. Q2, Q1 vs. Q3, and Q1 vs. Q4, showed higher SBP in the higher Na:K ratio groups while the remaining pairwise comparisons showed no significance in the SBP differences. Q2, Q3, and Q4 had higher SBP by 1.09 (*p* < 0.05), 0.88 (*p* < 0.05), 1.41mmHg (*p* < 0.01) compared to Q1, respectively. In comparisons of DBP, two pairwise comparisons, Q1 vs. Q2 and Q1 vs. Q4, showed higher DBP in the higher Na:K ratio groups by 0.83 (*p* < 0.01) and 0.92 (*p* < 0.01), respectively. From the significant differences in SBP and DBP between Q1 and Q2, we could infer 2.37 and 1.80 mmHg increases in SBP and DBP, respectively, in response to one unit of change in the Na:K ratio. Therefore, the results showed that the Na:K ratio also had a significant effect on the blood pressure, and especially, Q1 had significantly lower blood pressure compared to the other quartiles.

## 4. Discussion and Conclusions

We found that the Na:K ratio had a significant effect on hypertension prevalence and blood pressure. Groups with higher Na:K ratio had higher hypertension prevalence rates; there was no evidence that usual Na:K ratio groups, Q2 or Q3, had lower hypertension risk than the lower Na:K ratio group, Q1. Furthermore, groups with higher Na:K ratio had consistently higher SBP than the lower Na:K ratio group, Q1. That is, the lower ratio group (Q1) had the lowest risk in blood pressure, and the blood pressure results showed almost a linear dose-response relationship between the Na:K ratio and SBP. These results deny the possibility of an existence of a U-shaped relationship between the Na:K ratio and hypertension and between the Na:K ratio and blood pressure.

The mechanisms by which sodium and potassium affect blood pressure are multiple. In salt-sensitive individuals, salt ingestion causes sodium and water retention and extracellular volume expansion, which results in the release of substances increasing heart and blood vessel contraction and affecting renin-angiotensin-aldosterone system [42,43]. Potassium increases urinary sodium excretion which diminishes body sodium. In addition, potassium is thought to induce vascular smooth muscle relaxation and thus decrease peripheral resistance [44]. The blood pressure lowering effects of potassium intake were greater in individuals ingesting higher sodium intakes [45], and the Na:K ratio had a stronger effect on the risk of cardiovascular disease than sodium or potassium alone [46]. Therefore, people should be more concerned about the Na:K ratio in their diet than the level of sodium or potassium individually. In the present study, the low Na:K ratio group (Q1) consumed higher amounts of fruits and potatoes and lower amounts of cereals, salty vegetables, meats, and meat products than the high Na:K ratio group (Q4). Recommending a diet with sufficient fresh vegetables, fruits, and low fat dairy, which is similar to DASH diet [17], is a good way to achieve low Na:K ratio. This dietary pattern is also known as a ‘healthy diet’ or ‘prudent diet’ in many studies, such as Hu et al. [28], Baik et al. [47], Newby et al. [48], etc. Low Na:K ratio in Korean diet can be achieved by a comprehensive community based approach and in cooperation with the food industry as shown in the experiences of Finland [49] and England [50].

Our result is consistent with the results of some observational studies in various countries that Na:K ratio has a significant effect on hypertension prevalence and/or blood pressure [9,11,12,51]. Du et al. [9] found a strong association between the Na:K ratio and hypertension among Chinese adults. Zhang et al. [11] was very similar to our work in that they used pooled cross-sectional data and sodium intake data form 24 h recalls. They found the difference in hypertension prevalence rates only in their lowest and highest quartile comparison among US adults; however, they did not find the association between the Na:K ratio and diastolic blood pressure. Huggins et al. [12] found the association with systolic blood pressure but not with diastolic blood pressure among older Australian adults. Schröder et al. [51] also acknowledge the association between the Na:K ratio and diastolic blood pressure among Spanish population; however, they failed to detect the association between the Na:K ratio and systolic blood pressure in normotensive and non-medicated hypertensive subjects. All the observational studies except Du et al. showed some inconsistency in the association between the Na:K ratio and blood pressure. The most striking difference between these studies and ours is that their study population contained subjects with chronic disease.

The results of our study were obtained using the sound statistical approaches recommended by the American Heart Association Science Advisory. Especially, the effect of the Na:K ratio on blood pressure was obtained after removing almost all of the salt sensitive subjects by excluding the subjects taking hypertension medications. Even though the actual blood pressure difference in SBP and DBP from the pairwise comparisons was not huge, the small shift of whole blood pressure distribution could have a significant effect on the burden of hypertension [52].

However, our study has a few potential limitations. One limitation of our study is the use of cross-sectional data; assessments of nutrients intake and blood pressure were made at the same time. Therefore, our study cannot determine whether the nutrient intake preceded the blood pressure change and, hence, whether the nutrient intake caused the blood pressure change. However, the propensity score matching can provide a useful approximation of random trial and an approximate effect of the Na:K ratio on health outcomes [32].

The second potential limitation is that our study used the Na:K ratio based on the food intake survey, because the incorrect measure of sodium and potassium intake could bias the results. Our result regarding blood pressure favors lower Na:K ratio, and Na:K ratio is positively associated with blood pressure increases even if the quantity of blood pressure increase is small. Thus, the direction of association seems reasonable, but the small quantity of blood pressure increase could be a limitation. Since errors in sodium intake measurement could attenuate the association between sodium intake and health risk [22], larger blood pressure increase would have been obtained if multiple, nonconsecutive 24 h urine excretion data were used. The golden rule for urine sample collection is to collect nonconsecutive, multiple days, 24 h urine; however, such a method is impractical for large-scale surveys [10]. Another limitation is that the sodium or potassium specific effect on hypertension prevalence or blood pressure was not available because our model used the Na:K ratio variable.

Our study contributes to the literature by providing evidence of an association between the Na:K ratio and the hypertension prevalence and blood pressure based on the sound statistical method. The relationship between sodium intake and cardiovascular diseases and/or stroke has become more plausible than ever with this evidence since hypertension is a direct risk factor of those diseases. Moreover this study provides a quantitative effect of the Na:K ratio on the hypertension prevalence and blood pressure from a large pool of subjects. However, there exists large difference in sodium sensitivity among different ethnic groups as suggested by Franco and Oparil [53], therefore, a cautious interpretation of our results is advised.

Further research is needed to determine whether the results will hold up after controlling for genetic factors since more results are being reported that assess the effect of not only sodium intake but genetic variations on the blood pressure [54,55]. A similar approach to cohort data is needed to analyze the exact causal effect.

## Figures and Tables

**Table 1:** Definitions and descriptive statistics of study population.

Variables	Definitions	Mean ^a^ (*n* = 9424)
Sodium intake	The quantity of sodium intake per day (mg/day)	4533.17 ± 30.24
Potassium intake	The quantity of potassium intake per day (mg/day)	3104.64 ± 15.97
Sodium to potassium ratio	The quantity of sodium intake divided by the quantity of potassium intake (Na/K)	1.54 ± 0.01
Hypertension (%)	1 if systolic blood pressure ≥140 mmHg, diastolic blood pressure ≥90 mmHg or currently taking hypertension medications, 0 otherwise.	19.56
Body mass index	The body weight divided by the square of height (kg/m^2^)	22.87 ± 0.03
Walking exercise	Time spent walking per day (hours/day)	0.67 ± 0.01
Smoking status (%)	1 if a subject is currently smoking, 0 otherwise	14.51
Drinking status (%)	1 if a subject drinks a glass or more/month for the last 1 year, 0 otherwise	53.28
Daily stress level (%)	1 if a subject reports “high” or “very high” levels of usual stress, 0 otherwise	24.49
Use of nutrition label (%)	1 if a subject reads the nutrition label, 0 otherwise	30.19
Family history of hypertension (%)	1 if a subject’s father, mother, or siblings have ever been diagnosed with hypertension, 0 otherwise	37.37
Female (%)	1 if sex is female, 0 otherwise	68.19
Age	Age (years)	46.12 ± 0.16
Marital status (%)	1 if unmarried, 0 otherwise	15.86
Manual worker (%)	1 if a subject has a manual job, 0 otherwise	22.50
Household Income	Monthly household income (million won/month)	446.57 ± 8.05
Education	Schooling year: elementary school graduation = 6, middle school graduation = 9, high school graduation = 12, university = 16	12.30 ± 0.04

^a^ All values for continuous variables were expressed as the mean ± standard deviation or the percentage for categorical variables.

**Table 2:** Summary statistics of the study population divided into quartiles of the sodium to potassium ratio.

Variables	Q1	Q2	Q3	Q4	*p*-Difference	*p*-Trend ^a^
*n*	2356	2356	2356	2356		
(Sodium and potassium variables)						
Sodium intake (mg/day)	2578.22	3783.20	4770.62	7000.64	0.000 ^b^	(+) 0.000
Potassium intake (mg/day)	3638.99	3113.01	2919.98	2746.57	0.000 ^b^	(−) 0.000
Sodium to potassium ratio (Na/K)	0.74	1.22	1.64	2.57	0.000 ^b^	(+) 0.000
(Health-related variables)						
Hypertension (%)	19.27	18.00	19.44	21.52	0.023	(+) 0.025
Body mass index (kg/m^2^)	22.88	22.78	22.87	22.93	0.438	(+) 0.668
Walking exercise	0.71	0.65	0.67	0.67	0.270	(−) 0.001
Smoking status (%)	9.00	13.92	16.72	18.38	0.000	(+) 0.000
Drinking status (%)	44.57	54.07	57.60	56.88	0.000	(+) 0.000
Daily stress level (%)	22.71	23.64	25.47	26.15	0.022	(+) 0.002
Use of nutrition label (%)	31.96	30.35	31.62	26.83	0.000	(−) 0.001
Family history of hypertension (%)	39.26	37.61	38.37	34.25	0.002	(−) 0.001
(Socio-demographic variables)						
Female (%)	77.42	68.42	64.52	62.39	0.000	(−) 0.000
Age (year)	48.17	45.62	44.70	45.99	0.000	(−) 0.000
Marital status (%)	13.88	15.15	17.83	16.60	0.001	(+) 0.001
Manual worker (%)	18.97	21.86	23.09	26.06	0.000	(+) 0.000
Household income (million won/month)	4.53	4.52	4.54	4.27	0.588	(−) 0.000
Education (year)	12.15	12.39	12.49	12.15	0.001	(+) 0.757
(Other dietary variables)						
Energy intake (kcal/day)	1886.51	1992.44	2025.98	2016.89	0.000	(+) 0.000
Protein intake (g/day)	64.76	71.97	74.26	73.96	0.000	(+) 0.000
Fat intake (g/day)	36.54	43.45	45.81	45.01	0.000	(+) 0.000
Carbohydrate intake (g/day)	321.50	316.61	310.31	309.18	0.001	(−) 0.007
Fiber intake (g/day)	9.43	7.17	6.86	7.12	0.000	(−) 0.000
Cereals and cereal products (kcal/day)	891.22	1008.12	1029.62	1083.04	0.000	(+) 0.000
Potatoes and starches (kcal/day)	86.91	41.56	32.34	19.73	0.000	(−) 0.000
Vegetables (kcal/day)	83.87	87.56	88.01	91.44	0.001	(+) 0.000
Fruits (kcal/day)	174.04	103.45	75.99	57.96	0.000	(−) 0.000
Meat and meat products (kcal/day)	133.22	189.94	208.58	199.43	0.000	(+) 0.000

^a^ Nonparametric test for trend was estimated with Stata’s nptrend command; ^b^ Adjusted for energy intake.

**Table 3:** Average treatment effects (ATT) of the sodium to potassium ratio on hypertension prevalence and nutrition intake for six pairwise comparisons using propensity score matching.

Variables	Q1 vs. Q2		Q1 vs. Q3		Q1 vs. Q4		Q2 vs. Q3		Q2 vs. Q4		Q3 vs. Q4	
	Control	Treatment	Control	Treatment	Control	Treatment	Control	Treatment	Control	Treatment	Control	Treatment
*n*	1970	1970	1897	1897	1859	1859	2188	2188	2103	2103	2183	2183
Hypertension prevalence (%)	17.56	18.58	17.13	19.87	18.77	22.22	17.64	20.11	18.12	21.07	20.43	19.65
ATT (% point)	1.02		2.74		3.44		2.47		2.95		−0.78	
*p*-value ^a^	0.204		0.015		0.005		0.019		0.008		0.260	
Change in hypertension prevalence per unit change of sodium to potassium ratio (% points)	2.16		3.08		1.89		5.88		2.18		−0.84	
Sodium to potassium ratio (Na/K)	0.74	1.21	0.74	1.64	0.74	2.56	1.22	1.63	1.22	2.57	1.64	2.56
Sodium intake (mg/day)	2588.94	3692.91	2610.23	4628.49	2594.76	6737.10	3813.46	4725.11	3799.56	6932.19	4762.82	7023.80
Potassium intake (mg/day)	3641.10	3050.27	3641.29	2833.01	3629.11	2652.12	3139.32	2893.91	3126.56	2721.18	2914.06	2768.39

^a^ *p*-values are for 1-tailed *t*-tests regarding the significance of ATTs.

**Table 4:** Statistics for balance tests before and after propensity score matching.

**Variables**	**Q1 vs. Q2**				**Q1 vs. Q3**				**Q1 vs. Q4**			
	**% Bias**		***p*-Value ^a^**		**% Bias**		***p*-Value**		**% Bias**		***p*-Value**	
	**Before**	**After**	**Before**	**After**	**Before**	**After**	**Before**	**After**	**Before**	**After**	**Before**	**After**
Body mass index	−3.0	−1.6	0.295	0.625	−0.3	−0.7	0.905	0.824	1.8	0.3	0.544	0.938
Walking exercise	−5.5	0.4	0.058	0.891	−3.7	0.6	0.202	0.850	−4.1	0.1	0.164	0.971
Smoking status	15.5	−0.6	0.000	0.834	23.2	−2.5	0.000	0.395	27.5	−2.7	0.000	0.369
Drinking status	18.2	−3.4	0.000	0.293	26.3	−1.9	0.000	0.559	24.8	2.1	0.000	0.533
Daily stress level	2.2	−0.8	0.448	0.791	6.5	1.4	0.027	0.676	8.0	0.3	0.006	0.940
Use of nutrition label	−3.5	3.5	0.232	0.274	−0.7	0.3	0.802	0.918	−11.3	1.9	0.000	0.565
Family history of hypertension	−3.4	0.8	0.243	0.793	−1.8	1.4	0.530	0.665	−10.4	1.6	0.000	0.634
Female	−20.3	4.6	0.000	0.141	−28.7	2.5	0.000	0.435	−33.2	3.1	0.000	0.334
Age	−17.2	0.2	0.000	0.945	−23.4	1.7	0.000	0.598	−14.3	−1.6	0.000	0.627
Marital status	3.6	−0.3	0.215	0.928	10.8	−2.0	0.000	0.532	7.6	0.6	0.009	0.853
Manual worker	7.2	−0.4	0.014	0.904	10.1	0.1	0.001	0.968	17.0	−2.7	0.000	0.398
Household Income	−0.2	1.2	0.952	0.708	0.1	−1.0	0.982	0.766	−3.7	3.1	0.205	0.303
Education	6.7	−1.3	0.021	0.691	9.3	−1.1	0.001	0.725	−0.0	1.7	0.997	0.613
	Pseudo R^2^		*p* > LR^b^ (χ^2^^)^		Pseudo R^2^		*p* > LR (χ^2^^)^		Pseudo R^2^		*p* > LR (χ^2^^)^	
Over-all balance tests	0.022	0.001	0.000	0.979	0.037	0.000	0.000	1.000	0.041	0.001	0.000	0.993
**Variables**	**Q2 vs. Q3**				**Q2 vs. Q4**				**Q3 vs. Q4**			
	**% Bias**		***p*-Value ^a^**		**% Bias**		***p*-Value**		**% Bias**		***p*-Value**	
	**Before**	**After**	**Before**	**After**	**Before**	**After**	**Before**	**After**	**Before**	**After**	**Before**	**After**
Body mass index	2.7	−0.6	0.362	0.837	4.7	−2.2	0.107	0.469	2.1	−0.5	0.475	0.856
Walking exercise	1.8	−0.6	0.541	0.841	1.3	1.4	0.655	0.643	−0.4	1.6	0.884	0.602
Smoking status	7.8	−3.0	0.008	0.301	12.1	−7.4	0.000	0.011	4.4	−2.0	0.135	0.495
Drinking status	7.1	−3.5	0.015	0.247	5.6	−0.5	0.053	0.877	−1.5	2.8	0.617	0.358
Daily stress level	4.2	−2.8	0.146	0.358	5.8	0.7	0.047	0.830	1.6	−1.6	0.594	0.603
Use of nutrition label	2.8	−1.2	0.345	0.696	−7.8	0.3	0.007	0.919	−10.6	0.4	0.000	0.894
Family history of hypertension	1.6	−1.3	0.589	0.663	−7.0	−2.5	0.016	0.424	−8.6	1.0	0.003	0.754
Female	−8.3	3.7	0.005	0.221	−12.7	4.7	0.000	0.123	−4.4	0.5	0.130	0.875
Age	−6.3	3.2	0.031	0.284	2.5	1.5	0.400	0.638	8.5	−5.0	0.003	0.091
Marital status	7.2	−5.2	0.013	0.078	3.9	−0.8	0.175	0.799	−3.3	1.3	0.263	0.662
Manual worker	2.9	−0.7	0.312	0.828	9.9	−3.3	0.001	0.272	6.9	−5.0	0.018	0.092
Household Income	0.2	0.2	0.941	0.945	−3.1	0.0	0.287	1.000	−3.4	2.2	0.248	0.425
Education	2.7	−0.5	0.347	0.881	−6.8	0.4	0.020	0.896	−9.4	5.7	0.001	0.055
	Pseudo R^2^		*p* > LR (χ^2^^)^		Pseudo R^2^		*p* > LR (χ^2^^)^		Pseudo R^2^		*p* > LR (χ^2^^)^	
Over-all balance tests	0.004	0.001	0.036	0.900	0.007	0.002	0.000	0.638	0.004	0.001	0.008	0.790

^a^ *p*-values are for the test of equality of sample means between the control and treatment groups; ^b^ LR stands for the likelihood ratio test and test statistic has χ^2^ distribution.

**Table 5:** Average treatment effects (ATT) of the sodium to potassium ratio on blood pressure for six pairwise comparisons using propensity score matching after excluding hypertension patients

Models	Blood Pressure (mm Hg)		ATT (mm Hg) (Standard Error)	*p*-Value ^a^
	Control	Treated		
Systolic blood pressure				
Q1 vs. Q2	111.34	112.43	1.09 (0.51)	0.017
Q1 vs. Q3	111.05	111.93	0.88 (0.52)	0.046
Q1 vs. Q4	111.28	112.70	1.41 (0.54)	0.005
Q2 vs. Q3	112.14	112.31	0.17 (0.49)	0.362
Q2 vs. Q4	112.48	112.76	0.28 (0.50)	0.292
Q3 vs. Q4	112.93	112.60	−0.33 (0.49)	0.255
Diastolic blood pressure				
Q1 vs. Q2	72.51	73.34	0.83 (0.32)	0.005
Q1 vs. Q3	72.44	72.63	0.19 (0.33)	0.284
Q1 vs. Q4	72.55	73.46	0.92 (0.34)	0.004
Q2 vs. Q3	73.44	73.11	−0.33 (0.31)	0.141
Q2 vs. Q4	73.58	73.64	0.06 (0.32)	0.426
Q3 vs. Q4	73.44	73.74	0.30 (0.32)	0.173

^a^ *p*-values are for 1-tailed t-tests regarding the significance of ATTs.
